# Characterization of Psychopathology in Latin American Adolescents Using a Web-Based Screening Tool: Cross-Sectional Study

**DOI:** 10.2196/57038

**Published:** 2024-08-08

**Authors:** Susana Campos, Daniel Nuñez, J Carola Pérez, Jo Robinson

**Affiliations:** 1 Center of Applied Psychology Faculty of Psychology University of Talca, campus Talca Talca Chile; 2 Millennium Nucleus to Improve the Mental Health of Adolescents and Youths (Imhay) Santiago Chile; 3 Center of Cognitive Sciences Faculty of Psychology University of Talca Talca Chile; 4 Faculty of Psychology University of Talca, Campus Talca Talca Chile; 5 Instituto de Bienestar Socioemocional Facultad de Psicología Universidad del Desarrollo Santiago Chile; 6 Orygen Parkville, Victoria Australia; 7 Centre for Youth Mental Health The University of Melbourne Parkville, Victoria Australia

**Keywords:** web-based screening, adolescents, psychopathology, suicidal ideation, early detection, detection, screening, teens, youths, suicide, mental health, screening tool, Latin American, Latino, psychiatric, psychiatric symptoms, psychological risk

## Abstract

**Background:**

Mental health problems and suicide ideation are common in adolescents. Early detection of these issues could prevent the escalation of mental health–related symptoms in the long term. Moreover, characterizing different profiles of prevalent symptoms in conjunction with emotional regulation strategies could guide the design of specific interventions. The use of web-based screening (WBS) tools has been regarded as a suitable strategy to timely detect symptomatology while improving the appeal, cost, timeliness, and reach of detection in young populations. However, the evidence regarding the accuracy of these approaches is not fully conclusive.

**Objective:**

The study aims (1) to examine the capability of a WBS to identify adolescents with psychiatric symptoms and suicidality and (2) to characterize the mental health profiles of a large sample of adolescents using WBS.

**Methods:**

A total of 1599 Latin American Spanish-speaking adolescents (mean age 15.56, SD 1.34 years), consisting of 47.3% (n=753) female, 98.5% Chilean (n=1570), and 1.5% Venezuelan (n=24) participants, responded to a mental health WBS. A randomized subsample of participants also responded to the Mini International Neuropsychiatric Interview for Children and Adolescents (MINI-KID). McNemar *χ*^2^ and receiver-operating characteristic curves tested the detection accuracy of WBS contrasted with the MINI-KID. Latent profile analyses explored the symptomatic and emotional regulation profiles of participants.

**Results:**

Both measures showed an adequate level of agreement (area under the curve per symptom domain ranging from 0.70 to 0.89); however, WBS yielded a higher prevalence than MINI-KID for all psychiatric symptoms, except suicide ideation and depression. Latent profile analyses yielded 4 profiles—one of them presented elevated psychopathological symptoms, constituting 11% of the sample (n=175). Rumination (odds ratio [OR] 130.15, 95% CI 51.75-439.89; *P*<.001), entrapment (OR 96.35, 95% CI 29.21-317.79; *P*<.001), and defeat (OR 156.79, 95% CI 50.45-487.23; *P*<.001) contributed significantly to the prediction of latent profile memberships, while cognitive reappraisal did not contribute to the prediction of any latent profile memberships, and expressive suppression was only associated to profile-2 membership.

**Conclusions:**

WBS is acceptable for the timely detection of adolescents at risk of mental health conditions. Findings from the symptomatic and emotional regulation profiles highlight the need for comprehensive assessments and differential interventions.

## Introduction

Mental disorders and suicidal-related behaviors (SRBs) are prevalent in adolescents [1,2] and represent a major global health burden [3]. Developing and reinforcing evidence-based preventive approaches to actively identify this phenomenon in adolescents is a well-recognized need, as it could lead to timely interventions [4,5]. This is particularly relevant for adolescents from Latin American countries, where the gaps for mental health treatment are very high [6] and SRBs are one of the greatest health concerns [7,8]. Barriers to accessing mental health support, combined with a preference for self-reliance when experiencing psychological and emotional problems [9], may lead youth to search for alternatives to more traditional means of obtaining care, such as digital support or information concerning their mental health state [10].

Moreover, a relevant proportion of school-aged youth face mental health problems that go undetected [11]. Digital screening technologies could help overcome this obstacle, as they are currently regarded as suitable for assessing a broad range of psychopathological variables while improving the appeal, cost, timeliness, and reach of detection programs and preventive strategies [12-14]. Overall, digital mental health screening approaches have been reported as a preferred method for self-disclosure, which has been well-received among young people [15]. Some web-based screening (WBS) tools for mental health symptoms and SRBs have been recently and successfully developed for different populations [16], such as individuals in community settings [17], youth in emergency departments [18], university students [19], and school-aged Latin American youth [17].

Moreover, digital approaches seem to be promising for the implementation of current tools of psychological risk stratification that guide decision-making for care pathways, such as the clinical staging model [20]. This model uses a transdiagnostic perspective that allows for the comprehension of the interrelation existent between psychopathology [21], suicidality [22], and other mechanisms (ie, emotional regulation strategies). Accordingly, this approach suggests that the assessment of a broad range of clinical and subclinical symptoms, along with other individual factors, is helpful in identifying different symptomatic profiles in young people and ultimately improves early detection and intervention strategies [23]. One of the statistical techniques suitable to integrate this perspective is latent profile analysis (LPA), a latent variable modeling approach [24,25] that allows for classifications of individuals into latent subgroups based on their scoring patterns on a range of manifest variables.

In this context and based on literature encouraging the use of digital technology to improve the screening for mental disorders and suicide ideation (SI) [19], we examined the capability of a WBS to identify adolescents and school-aged youth with high levels of psychiatric symptoms and SI. We expected to find that the WBS detects a similar proportion of at-risk individuals compared to a standardized clinical interview. Subsequently, we conducted an LPA to characterize the mental health profiles of a large sample of adolescents using WBS. We hypothesized that a small percentage of adolescents could display a profile characterized by severe psychiatric symptomatology and SI. Additionally, we explored whether emotional regulation strategies could predict the adscription to the identified symptomatologic profiles. We hypothesized that adolescents with higher levels of symptoms could mostly use emotional regulation strategies associated with experiences of psychological distress (eg, rumination, emotional suppression, defeat, and entrapment) than adolescents with lower or without symptoms who use adaptive strategies (eg, cognitive reappraisal).

## Methods

### Participants

We conducted a cross-sectional study with 1599 Latin American adolescents recruited between April and September 2019 in 11 urban public secondary schools in Chile. The inclusion criteria were that the students and their caregivers voluntarily agreed to participate in the study and signed written informed consent. We excluded 5 individuals who wrongly defined their ages (out of the range of 12-19 years). We performed the analyses with a final sample of 1594 adolescents (mean age 15.56, SD 1.34 years), where 47.3% (n=753) was female. Of the total sample, 98.5% was Chilean (n=1570), and 1.5% Venezuelan (n=24).

### Measures

#### SI Measurement

We used 7 items of the Columbia-Suicide Severity Rating Scale [26], adapted as a self-report questionnaire in the Chilean population [27]. The severity of lifetime SI was rated on a 7-item binary scale (1=yes and 0=no). All items were added to obtain a total score. Adolescents who denied ideation received a score of 0. Scores of 3 and over were deemed as “at-risk.” The internal consistency of SI was good for the current population (Cronbach α=0.88; McDonald ω=0.90).

#### Criteria and Severity of Depressive Disorder and Symptoms

We used the Patient Health Questionnaire [28] for adolescents [29]. It uses a 4-point scale ranging from 0=not at all to 3=nearly every day. Overall scale scores are computed as a sum of the 9 items (possible range 0-27). Scores can be divided into 5 severity categories: none, mild, moderate, moderately severe, and severe [30]. This scale was adapted to Chilean adolescents [31]. In our sample, its reliability was good (α=0.89; ω=0.90).

#### Criteria and Severity of Generalized Anxiety Disorder and Symptoms

We used the Generalized Anxiety Disorder-7 (GAD-7) [32], a 7-item self-report questionnaire where respondents indicate how often they have been bothered by 7 core symptoms of the generalized anxiety disorder. The answers are rated on a 4-point scale as 0=not at all, 1=several days, 2=more than half the days, and 3=nearly every day. GAD-7 sum scores range from 0 to 21. The cutoff for moderate anxiety is 10 points, and 15 or more points represent severe anxiety symptoms (ASs). This scale was validated in the Chilean population [33]. In our sample, its reliability was excellent (α=0.93; ω=0.93).

#### Psychotic Experiences

We addressed psychotic experiences (PE) with the Chilean adaptation of the Community Assessment of Psychic Experiences-Positive [34,35]. Responses to the 15 items range from 1=never to 5=nearly always and are summed to obtain a total score. The Community Assessment of Psychic Experiences-Positive addresses paranoid ideation (5 items), bizarre experiences (7 items), and perceptual abnormalities (3 items). The reliability in our sample was excellent for the complete measure (α=0.901; ω=0.903) and good for its subscales (paranoid ideation: α=0.917; ω=0.92; bizarre experiences: α=0.846; ω=0.853; and perceptual abnormalities: α=0.795; ω=0.815).

#### Posttraumatic Stress Disorder

The brief posttraumatic stress disorder (PTSD) scale, Chilean adaptation [36] is a list of PTSD symptoms (5 items) and complex PTSD symptoms (3 items). The measure format is a 5-point Likert scale (1=never to 5=very often). The total score is estimated by adding the score obtained in each item. In our sample, its reliability was excellent (α=0.921; ω=0.922).

#### Mini International Neuropsychiatric Interview for Children and Adolescents

The Mini International Neuropsychiatric Interview for Children and Adolescents (MINI-KID) version 6.0 in Spanish [37] is a short structured diagnostic interview for people aged 4-17 years. We assessed the presence of the 24 specific *Diagnostic and Statistical Manual of Mental Disorders, Fourth Edition* child and adolescent psychiatric disorders it encompasses, as well as the suicide risk. The interview was led and coded by trained psychologists, who were blind to the results of the WBS at this stage.

### Procedure

We first developed a web-based platform for screening purposes. This WBS allows the programming of questionnaires, defines custom cutoff points for each, and identifies those users who are above the cutoff points as being at risk. All public schools in the central Maule region that had secondary education were invited to participate, and 11 agreed. Once written informed consent was obtained from students and their caregivers, participants completed a series of self-administered questionnaires included in the WBS on desktops in their school laboratories, while under the supervision of trained psychologists. Based on WBS alerts, psychologists conducted feedback interviews with adolescents showing high SI and their caregivers within 48 hours after the detection. The purpose was to assess the current risk, to alert caregivers, and to offer psychological intervention where appropriate. These participants were referred to primary care centers, where a protocol for managing SI defined by the Chilean Ministry of Health was applied when possible. To expedite the mental health referrals, 5 primary care health centers were contacted and agreed to participate in the study, providing psychotherapy to the adolescents identified as being at risk. Additionally, in a simple random subsample of 217 students, we assessed psychiatric symptoms through the MINI-KID within a month after the completion of the WBS. A computer-based number randomization was used to establish this subsample, through simple random sampling.

### Data Analysis

Descriptive statistics were calculated, followed by an assessment of the agreement between the WBS and the MINI-KID, comparing respective prevalences using the McNemar *χ*^2^. Additionally, we used a series of receiver-operating characteristic curves. Specifically, we tested agreement on SI, depressive symptoms (DS), PTSD, AS, and PE. Area under the curve (AUC) values were categorized as slight (0.50-0.59), fair (0.6-0.69), moderate (0.7-0.79), substantial (0.8-0.89), and almost perfect (>0.9) [38]. All analyses were run with SPSS (version 18, IBM Corporation).

The identification of profiles was performed through LPA. In LPA, 2 sets of parameters are of interest: (1) latent profile membership probabilities (ie, prevalence) that describe the distribution of profiles and (2) item-response means (and variances) that provide profile-specific means (and variances) based on symptomatology. Item-response means are used to interpret and label the profiles.

The model was estimated by the maximum likelihood restricted method using Mplus (version 8.6; Muthén & Muthén) [39]; estimation and model identification for all LPA models were checked using 1000 initial stage starts and 250 final stage starts. An assessment of solutions consisting of 1 to 7 profiles was performed considering indicators of model fit and interpretability of the clustering solution [40,41]. Theoretical and clinical interpretations were emphasized in the model selection because it is common in LPA that model fit indices continue to decrease as additional profiles are added [42]. Model fit indicators used were the Akaike information criterion [43], Bayesian information criterion [44], and sample size–adjusted Bayesian information criterion [45], on which lower values indicate better relative fit [46]. Additionally, the Lo-Mendell-Rubin adjusted likelihood ratio test and Vuong-Lo-Mendell-Rubin likelihood ratio test [47] were used to compare models that differ in the number of classes by indicating that the model with K – 1 classes should be rejected in favor of the model with K classes [48]. Entropy is reported as further evidence for profile selection, with 0.80 or greater providing evidence that profiles occur with minimal uncertainty [49,50]. A post hoc power analysis using Monte Carlo simulation was done. Following recommendations, 10,000 replications were estimated [51].

Between-profile differences on the continuous indicators based on posterior probabilities were investigated using a multivariate analysis of variance. Significant univariate effects were probed further using the Games-Howell post hoc test, which is robust to heterogeneity in the variance-covariance matrix.

Last, to explore whether emotional regulation strategies could predict the ascription to the previously identified symptomatologic profiles, multinomial logistic regression identified the profile membership on emotion regulation strategies (Multimedia Appendix 1).

### Ethical Considerations

This study and all associated documents were approved by the scientific ethics committee of the University of Talca (40.001.103-0; July 7, 2020), complying with American Psychological Association ethical standards in the treatment of the sample. Written informed consent was obtained from all participants and their legal guardians. This document emphasized the voluntary nature of the study and their ability to opt out at any moment. The collected data were completely deidentified and stored in secure servers before the analysis process began to ensure the privacy of participant data. Participants did not receive any type of compensation for their participation in the study at any point.

## Results

### Sample Characterization

Table 1 shows sociodemographic data and the prevalence of symptoms. Participants deemed as “at risk” (ie, moderate or high SI) had scores of 3 points and over on the Columbia-Suicide Severity Rating Scale. Accordingly, 269 (16.9%) of the 1594 participants (170/269, 63.2% female; 97/269, 36.1% male; and 2/269, 0.7% nonbinary) comprised the at-risk group. DS and AS were the most prevalent symptoms in both the total and the at-risk samples.

**Table 1:** Sociodemographic data and symptomatology in the WBS^a^ and MINI-KID^b^ samples.

Sociodemographic data and symptomatology		WBS					Clinical interview (MINI-KID)			
		Total cohort (n=1594)		At-risk^c^ cohort (n=269)		Total cohort (n=217)			At-risk^d^ cohort (n=53)	
		Mean (SD)	n (%)	Mean (SD)	n (%)	Mean (SD)		n (%)	Mean (SD)	n (%)
Age (years)		15.5 (1.3)	—^e^	15.8 (1.3)	—	15.5 (1.2)		—	15.4 (1.3)	—
Sex (female)		—	753 (47.3)	—	170 (63.2)	—		92 (42.4)	—	32 (60.4)
Fail grade		—	336 (21.1)	—	60 (22.3)	—		46 (21.2)	—	13 (24.6)
Previous psychiatric treatment		—	36.4 (36.4)	—	177 (65.8)	—		65 (30)	—	24 (45.3)
**Family configuration**										
	Biparental home	—	871 (54.7)	—	118 (44.2)	—		123 (57)	—	25 (47.2)
	Single parent home (mother)	—	575 (36.1)	—	111 (41.3)	—		70 (32.3)	—	22 (41.5)
	Living with other relatives	—	97 (6.1)	—	24 (8.9)	—		12 (5.5)	—	5 (9.4)
	Foster home	—	11 (0.7)	—	2 (0.7)	—		2 (0.9)	—	0
**Monthly house income**										
	Lesser minimum wage	—	749 (47)	—	119 (44.3)	—		109 (50.3)	—	46 (86.8)
	Greater minimum wage	—	843 (52.9)	—	889 (55.8)	—		108 (49.7)	—	7 (13.2)
Drug use		—	156 (9.8)	—	29 (10.8)	—		13 (6)	—	10 (18.9)
Alcohol use		—	312 (19.6)	—	100 (37.2)	—		32 (14.8)	—	12 (22.6)
Psychiatric treatment relative		—	456 (28.6)	—	113 (42)	—		49 (22.6)	—	19 (35.8)
Suicide attempts relative		—	162 (10.2)	—	68 (25.3)	—		22 (10.1)	—	10 (18.9)
Relative committed suicide		—	124 (7.8)	—	38 (14.1)	—		16 (7.4)	—	7 (13.2)
Depression		9.4 (6.8)	465 (29.2)	15.1 (5.8)	209 (77.7)	7.7 (7.4)		45 (20.7)	15.4 (8.6)	36 (67.6)
Anxiety		8.1 (5.3)	758 (47.6)	21.1 (8.4)	203 (75.5)	6.7 (5.3)		82 (37.8)	17.2 (8.9)	26 (48.6)
Posttraumatic stress		16.5 (7.7)	377 (23.7)	25.1 (7.6)	146 (54.3)	—		42 (19.4)	21.7 (9.5)	21 (40.5)
PTSD^f^		10.9 (5.2)	—	16.3 (5.1)	—	14 (7.7)		—	14.5 (6.5)	—
Complex PTSD		6 (3)	—	9.2 (3.1)	—	5.3 (3)		—	8.2 (3.6)	—
Psychotic experiences		25 (9.1)	334 (21)	34.1 (12)	145 (53.9)	23.1(10.8)		34 (15.7)	34 (15.1)	23 (43.2)
Paranoid ideation		9.7 (3.8)	260 (16.3)	11.3 (4.1)	123 (45.7)	8.7 (4.1)		30 (13.8)	12.6 (5.7)	18 (35.1)
Bizarre experiences		11 (4.7)	145 (9.1)	12.8 (5.5)	73 (27.1)	10.3 (5.4)		19 (8.8)	15.6 (7.8)	18 (35.1)
Perceptual anomalies		4.1 (2)	70 (4.4)	4.7 (2.4)	35 (13)	4.3 (3.2)		119 (5.5)	5.9 (3.4)	13 (24.3)

^a^WBS: web-based screening.

^b^MINI-KID: Mini International Neuropsychiatric Interview for Children and Adolescents.

^c^At-risk WBS sample: participants with scores of 3 or more on Columbia-Suicide Severity Rating Scale.

^d^At-risk MINI-KID sample: participants positive for suicidality according to the MINI-KID.

^e^Not applicable.

^f^PTSD: posttraumatic stress disorder.

### Agreement of Measures

Table 2 shows the symptom prevalence of the subsample of participants assessed by the WBS and the MINI-KID. McNemar tests yielded significant differences between the proportion of cases in all variables except for DS (SI: *P*=.03; DS: *P*>.99; all others: *P*<.001). Moreover, the WBS showed higher detection rates than the MINI-KID for all psychiatric symptoms, except for DS, where it identified a comparable number of cases, and SI, where it identified fewer cases. Moreover, we observed robust values of AUC for DS (AUC=0.818), PTSD (AUC=0.89), and PE (AUC=0.893). The AUC for SI was 0.782, and for AS, the AUC reached 0.702, which represents moderate criterion validity. Sensitivity and specificity levels for DS (86.4% and 57%, respectively), PTSD (80% and 82.9%), PE (83.3% and 78.6%), SI (73.6% and 74.4%), and AS (78.3% and 44%) were acceptable (Table S1 in Multimedia Appendix 1).

**Table 2:** Comparison of symptom prevalence in the subsample assessed by WBS^a^ and MINI-KID^b^ (n=217).

Symptoms	WBS			Clinical interview (MINI-KID)			McNemar test	
	n (%)	95% CI	n (%)		95% CI	Chi-square (*df*)		*P* value
Suicidal ideation	37 (17.1)	12.1-22.1	53 (24.4)		18.6-30.1	4.68 (1)		.03
Depression	45 (20.7)	15.2-26.1	44 (20.3)		15-25.5	000 (1)		>.99
Anxiety	57 (26.3)	20.4-32.1	23 (10.6)		6.5-14.6	20.07 (1)		<.001
Posttraumatic stress	34 (15.7)	10.8-20.5	5 (2.3)		0.3-4.2	25.29 (1)		<.001
Psychotic experiences	27 (12.4)	8-16.7	6 (2.8)		0.6-4.9	22.78 (1)		<.001

^a^WBS: web-based screening.

^b^MINI-KID: Mini International Neuropsychiatric Interview for Children and Adolescents.

### Profile Analysis

Table 3 summarizes the results of the model fit statistics of LPA. The results indicate that 4- and 5-profiles solutions are plausible. The 5-profile solution outperforms the 4-profile solution according to Lo-Mendell-Rubin adjusted likelihood ratio test and Vuong-Lo-Mendell-Rubin likelihood ratio test indicators (*P*<.05), but this solution has a lower level of entropy (0.79 vs 0.81). Also, it is not recommended to include clusters with less than 5% of participants, to avoid retention of “rare” or spurious clusters [41,52]. Additionally, the most significant decrease in the other model fit indicators occurred at the 4-profiles solution.

**Table 3:** Model fit statistics of latent profile analysis on adolescent’s symptomatology.

Model	AIC^a^	BIC^b^	SABIC^c^	Entropy	Smaller class (%)	VLMR^d^	LMR^e^
1-profile	20,863.34	20,927.83	20,889.71	—^f^	—	—	—
2-profiles	16,956.69	17,074.91	17,005.02	.91	31	<.001	<.001
3-profiles	15,902.69	16,074.66	15,973.00	.86	17	<.001	<.001
4-profiles^g^	15,581.35	15,807.05	15,673.63	.81	12	<.001	<.001
5-profiles	15,465.09	15,744.53	15,579.34	.79	5	<.05	<.05
6-profiles	15,342.05	15,657.24	15,460.28	.81	4	.14	.15
7-profiles	15,259.88	15,646.81	15,418.08	.81	2	.85	.85

^a^AIC: Akaike information criterion.

^b^BIC: Bayesian information criterion.

^c^SABIC: Sample-size adjusted Bayesian information.

^d^VLMR: Vuong-Lo-Mendell-Rubin adjusted likelihood ratio test.

^e^LMR: Lo-Mendell-Rubin adjusted likelihood ratio test.

^f^Not applicable.

^g^Latent profile analysis (LPA) model.

### Description of Profiles

**Figure 1:**
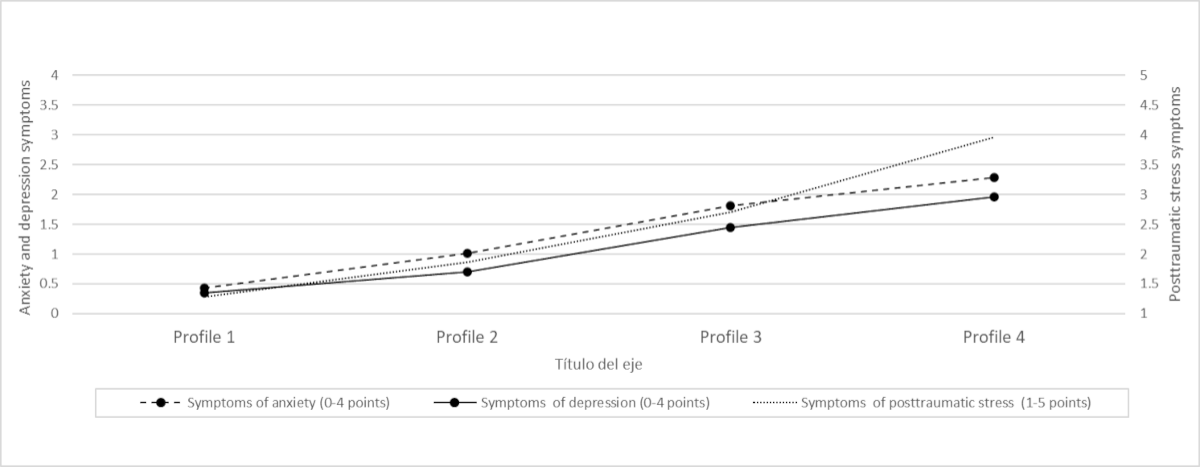
Latent profile characteristics for anxiety, depression, and posttraumatic stress disorder.

**Figure 2:**
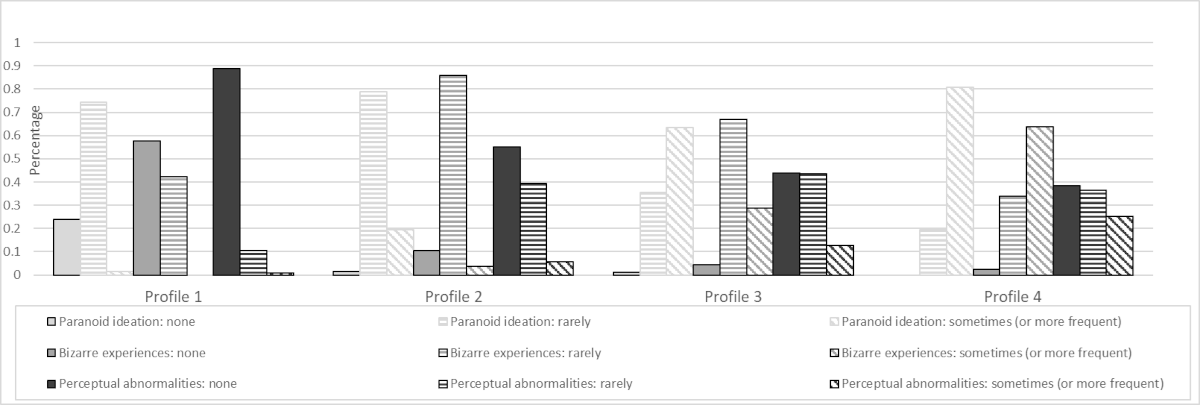
Latent profiles characteristics for psychotic experiences.

Profile characteristics are detailed in Table 4, Figure 1, Figure 2, and Table S2 in Multimedia Appendix 1. Multivariate analysis of variance results indicated that the 4 profiles differed significantly from one another on each of the continuous variables (Pillai trace=0.94; *F*_3,4761_=240.50; *P*<.001). Based on these results, clusters have been characterized as follows.

Profile 1 (no symptomatology; 32% of the subsample, [n=509]) showed very low depressive, anxious, and PTSD symptomatology and low probability of presenting PEs and reporting the absence of perceptual abnormalities (none=89%), none or scarce paranoid ideation (74%), and no risk of SI.

Profile 2 (low internalizing symptomatology; 34% of the subsample, [n=541]) presented low depressive, anxious, and PTSD symptomatology. In this profile, adolescents rarely reported having paranoid ideation (76%) and bizarre experiences (86%). Moreover, adolescents of this profile presented no risk of SI.

Profile 3 (low internalizing symptomatology and moderate posttraumatic stress symptomatology; 21% of the subsample, [n=334]) displayed low levels of anxiety and depression but moderate PTSD symptoms. Additionally, only 7% had a low probability of not reporting bizarre experiences. Adolescents in this group presented a high chance of not reporting SI, although this pattern is not as clear as in clusters 1 and 2.

Profile 4 (at-risk adolescents; 11% of the subsample, [n=175]) presented moderate depression symptoms, moderate anxiety, and high levels of PTSD symptoms. Moreover, this group has the highest probability of reporting more frequent bizarre experiences (72%) and paranoid ideation (90%). Also, adolescents of this profile have a higher probability of showing elevated SI (63%) compared to the other clusters.

The post hoc power results indicated that a minimum recommended power of 0.80 was obtained for most of the parameters estimated in the 4 profiles LPA with actual sample size, except for the bizarre experiences “sometimes (or more frequent)” category in profile 1 and profile 3.

**Table 4:** Profile description. Means and probabilities of latent class indicators (n=1591).

	LPA^a^ solution								Grand mean	
	Profile 1 (32%)		Profile 2 (34%)		Profile 3 (21%)		Profile 4 (11%)		Variable	*P* value
	Variable	*P* value	Variable	*P* value	Variable	*P* value	Variable	*P* value		
Symptoms of depression^b^; means comparison (probability)	0.37^c^ (0.35)	<.001	0.76^d^ (0.35)	<.001	1.33^e^ (0.35)	<.001	2.14^f^ (0.35)	<.001	0.92 (0.67)	<.001
Symptoms of anxiety^g^; means comparison (probability)	0.46^c^ (0.41)	<.001	1.06^d^ (0.41)	<.001	1.71^e^ (0.41)	<.001	2.33^f^ (0.41)	<.001	1.15 (0.75)	<.001
Symptoms of posttraumatic stress^g^; means comparison (probability)	1.31^c^ (0.53)	<.001	1.94^d^ (0.53)	<.001	2.85^e^ (0.53)	<.001	4.07^f^ (0.53)	<.001	2.18 (1.04)	<.001
SI^h^: none (≤2); categorical variables	0.99	<.001	0.93	<.001	0.67	<.001	0.37	<.001	0.83	<.001
SI: risk (≥3); categorical variables	0.00^c^	.198	0.07^d^	<.001	0.33^e^	<.001	0.63^f^	<.001	0.17	<.001
PI^i^: none and rarely; categorical variables	0.98	<.001	0.76	<.001	0.32	<.001	0.10	<.001	0.66	<.001
PI: sometimes (or more frequent); categorical variables	0.02^c^	.03	0.24^d^	<.001	0.67^e^	<.001	0.90^f^	<.001	0.34	<.001
BE^j^: none; categorical variables	0.55	<.001	0.10	<.001	0.07	<.001	0.01	.47	0.23	<.001
BE: rarely; categorical variables	0.45^c^	<.001	0.86^d^	<.001	0.61^e,f^	<.001	0.27^f^	<.001	0.60	<.001
BE: sometimes (or more frequent); categorical variables	0.00^c^	>.99	0.04^c,d^	<.001	0.32^c,e^	<.001	0.72^c,f^	<.001	0.17	<.001
PA^k^: None; categorical variables	0.89	<.001	0.52	<.001	0.42	<.001	0.29	<.001	0.59	<.001
PA: rarely; categorical variables	0.11^c^	<.001	0.42^d^	<.001	0.40^e^	<.001	0.33^f^	<.001	0.30	<.001
PA: sometimes (or more frequent); categorical variables	0.01^c^	.10	0.06^d^	<.001	0.18^e^	<.001	0.38^f^	<.001	0.11	<.001

^a^LPA: latent profile analysis.

^b^Likert scale (0=Never to 3=Nearly every day).

^c,d,e,f^Indicates statistical differences between profiles’ means or odds ratio (Table S3 in Multimedia Appendix 1).

^g^Likert scale (1=Never, 5=Very often). Categories for paranoid ideation, bizarre experiences, and perceptual abnormalities: none=1; rarely=1.1 to 2; sometimes (or more frequent)=3.1 to 5.

^h^SI: suicide ideation.

^i^PI: paranoid ideation.

^j^BE: bizarre experiences.

^k^PA: perceptual abnormalities.

### Exploring the Predictive Role of Emotional Regulation Strategies

When compared to the profile without symptomatology, the results indicated that cognitive reappraisal did not contribute to the prediction of latent profile memberships. Only the “low internalizing symptomatology” (profile 2) membership was associated with the expressive suppression emotion regulation strategy. Teenagers who say they often use expressive suppression had a higher chance of being in this latent profile (odds ratio [OR] 1.39, 95% CI 1.15-1.68; *P*<.001) compared to teens who did not have any symptoms.

In contrast, rumination, entrapment, and defeat contribute significantly to the prediction of latent profile memberships. If teenagers say they use these strategies more often, they are more likely to be in the profiles with some symptoms (profiles 2, 3, or 4) than in the first latent profile, which has no symptoms. For example, adolescents who were in the “at-risk profile” were more likely to ruminate (OR 130.15, 95% CI 51.75-439.89; *P*<.001), feel trapped (OR 96.35, 95% CI 29.21-317.79; *P*<.001), and being defeated (OR 156.79, 95% CI 50.45-487.23; *P*<.001; Table S3 in Multimedia Appendix 1).

## Discussion

### Principal Findings

First, we examined the capability of a WBS tool to identify adolescents with high levels of psychiatric symptoms and suicide risk in school-aged youth. Second, we characterized the mental health profiles of a large sample of adolescents using WBS.

The prevalence of SI, DS, and AS in our study was slightly lower than that found in previous studies [27], although it was relatively similar for PTSD [53]. Additionally, we found that SI was the only symptom domain to display a lower prevalence in the WBS than in the MINI-KID, which is contrary to previous findings [19]. Considering the significant short-term fluctuations of SI levels in this population [54], some of these differences could be explained by the assessment time gap between the WBS and MINI-KID (eg, 1- to 8-week gap). Additionally, the receiver-operating characteristic curves showed some level of agreement between the WBS and the MINI-KID for most of the assessed symptoms, particularly for depression symptoms, which have similar detection rates in both the WBS and MINI-KID. These findings show that, while not identical, there is some coincidence between measures. However, WBS appears to overdiagnose ASs. This is in accordance with previous evidence showing that the GAD-7 tends to show false positives in the nonclinical population [55].

According to our second aim, we found 4 distinct symptomatic profiles with the WBS tool. Most of them showed low symptom levels. However, we observed high levels of PTSD, AS, and DS in a small proportion of participants (“at-risk adolescents”; 11% of the sample). This group also showed a high probability of experiencing PE (mainly paranoid ideation and bizarre experiences). The higher presence of paranoid ideation followed by bizarre experiences mirrors the recent evidence found at the primary care level [56]. Regarding SI, adolescents in this cluster present the highest rates of SI in the sample. However, this pattern is not markedly clear within the profile. This phenomenon could be explained by the presence of high paranoid ideation that might have led to adolescents being reluctant to report SI in the digital questionnaire. This could also explain the lower rates of SI found in the WBS compared to the MINI-KID; adolescents may not disclose SI through a digital questionnaire (instead of a person), as they are unaware of the use that could be made of such information, and possibly fear being judged or labeled [57]. This is an implementation issue that would require further investigation to clarify; however, this escapes the aim of the current research. In cluster 3, adolescents display moderate levels of PTSD while having low levels of other symptomatology. This was a somewhat unexpected finding, particularly because PTSD symptoms are commonly found along somewhat similar levels of comorbid anxiety and DSs [58,59].

Regarding our findings on PE, the prevalence of these experiences (21%) was similar to those reported by Hafeez and Yung [60]; however, because self-report may lead to overestimations [61], further research is needed. We found a general pattern for paranoid ideation and bizarre experiences of PE: the endorsement rates of high scores systematically increased from clusters 1 to 4. This fits with prior research showing clear associations and overlaps between PE health conditions [62] and overall psychopathology [63] in the general population [64] and in clinical samples [56]. Moreover, these findings highlight both their role as early markers for psychopathology [65] and the need to regularly screen and deliver timely interventions for PE in young people [66,67]. As suggested by preliminary evidence, screening and referral of students were associated with a significant reduction in PEs [68].

Last, our exploratory examination of the role of emotional regulation strategies as potential predictors of the ascription to the identified profiles showed that rumination, defeat, and entrapment were associated with a higher probability of belonging to profiles with higher levels of symptomatology and SI (profiles 3 and 4). This fits with literature showing that these strategies are associated with the experience of emotional distress, depression, anxiety [69,70], PTSD symptoms [71], and SI [72]. By contrast, cognitive reappraisal was not associated with any symptom, and emotional suppression only differentiated between individuals presenting mild and no depression and ASs and was not a significant predictor of more severe symptom levels. This may seem counterintuitive, but the role of these 2 regulation strategies has not been completely clarified yet, as shown by a recent review and meta-analysis [73].

### Limitations

Our cross-sectional design does not allow us to establish causality among variables. Furthermore, the clinician-administered psychiatric interview could have increased social desirability in the answers [74], which in turn could have contributed to a lower prevalence of most disorders in the MINI-KID compared to the self-report [19]. Future research could attempt to comprehensively measure the psychopathological domains through a thorough self-report measure rather than a clinical interview. Additionally, social, and contextual variables (ie, perceived social support) were not included, and as their inclusion in future research could enrich study findings, it is advised. Last, although the application of the MINI-KID occurred anywhere from 1 week to 2 months after the use of WBS, longer times elapsed between measures could negatively affect the specificity and sensitivity reported for the web-based measure.

### Prevention Implications

WBS can improve the accessibility of evidence-based screening tools for the early detection of mental health problems in adolescents [75]. This can be particularly useful when access to health care can be restricted due to public health-related contingencies (eg, during the COVID-19 pandemic). Moreover, our finding revealing a relevant presence of common mental disorders and subthreshold psychopathological manifestations such as PEs in the cluster with higher levels of psychopathology, provides further support to include these phenomena in preventive programs for young people. This study is in line with the growing interest in the field to develop and test web-based tools for screening SRB and SI in young people [76], along with other mental health symptoms and psychological processes [77], diverging from the original approach of excluding individuals with suicidal risk from accessing updated screening techniques.

The identified profiles suggest that different interventions could be delivered to the adolescent population depending on their symptomatic levels or characteristics. For instance, universal programs to improve social and emotional well-being [78] might be useful for individuals in profiles 1 and 2. However, these interventions should be tailored and delivered with caution, as evidence has shown neutral to small effects [79]. Moreover, targeted (selective) interventions such as school-based programs for anxiety and DSs could be delivered for adolescents in profile 3, although further evidence is still needed for this approach, particularly regarding the temporal stability of their results [80].

Finally, given their symptomatology levels and fewer use of adequate emotional regulation strategies, indicated interventions and referrals to primary care centers for assessments and specialized care could be suitable for adolescents in profile 4.

In conclusion, we provide evidence about the suitability of a WBS as a tool for the timely detection of mental health risk, and the usefulness of developing symptomatic profiles in school-aged youth. Our findings support the use of WBS in this population but also suggest it is necessary to further improve the sensibility of digital screening tools.

## Appendix

Multimedia Appendix 1 Receiver operating characteristic for web-based screening (WBS) subsample (n=217).

## Abbreviations

AS: anxiety symptom
AUC: area under the curve
DS: depressive symptoms
GAD-7: Generalized Anxiety Disorder-7
LPA: latent profile analysis
MINI-KID: Mini International Neuropsychiatric Interview for Children and Adolescents
OR: odds ratio
PE: psychotic experience
PTSD: posttraumatic stress disorder
SI: suicide ideation
SRB: suicidal-related behavior
WBS: web-based screening

## Data Availability

The data sets generated or analyzed during this study are available from the corresponding author on reasonable request.
