# Supplementary material for: Characterization of Psychopathology in Latin American Adolescents Using a Web-Based Screening Tool: Cross-Sectional Study
Source: JMIR Form Res. 2024 Aug 8;8:e57038. doi: 10.2196/57038 (PMC11342013; doi:10.2196/57038)
Supplement: Multimedia Appendix 1 [file formative_v8i1e57038_app1.pdf]

Supplementary Material

Supplementary Table 1: Receiver operating characteristic for WBS subsample (n = 217)

	Cutoff point	SN (%)	PPV (%)	SP (%)	NPV (%)	AUC
SI	3	73.6	90.2	74.4	39.6	.782
Depression	4.5	86.4	89.5	57	59.1	.818
Anxiety	10.5	78.3	78.2	44	60.9	.702
PTSD	20.5	80	85.7	82.9	80	.890
PE	25.5	83.3	85.7	78.6	66.7	.893

Note: SN= sensitivity; SP= specificity; PPV= positive predictive value; NPV= negative predictive value.

Measures for supplementary analysis 2:

Defeat and entrapment (DE): The 8–item Short Defeat and Entrapment Scale (SDES; Griffiths et al., 2015; Martini, 2015), comprises four items assessing perceptions of defeat (e.g., "I feel defeated by life") and four items assessing perceptions of entrapment (e.g., "I would like to get away from who I am and start again"). Items range from 0= "not at all like me" to 4= "extremely like me". In our sample, its reliability was excellent (α=.92; ω=.93).

Emotion regulation (ER): The Emotional Regulation Questionnaire (ERQ; Gross & John, 2003; Cabello et al., 2013), assesses two ER strategies: cognitive reappraisal (REAPR; 6 items) and emotional suppression (SUPR; 4 items). Items range from 1 ("completely disagree") to 7 ("completely agree"). Its reliability was good (α=.82; ω=.83).

Rumination (R): We assessed ruminative thoughts by a 4-item short version of the Ruminative Responses Scale (Nolen-Hoeksema et al., 1999). Respondents rated each item on a scale from 1 ("almost never") to 4 ("almost always"). The scale showed a good reliability (α=.87; ω=.87).

References:

Griffiths, A. W., Wood, A. M., Maltby, J., Taylor, P. J., Panagioti, M., & Tai, S. (2015). The development of the Short Defeat and Entrapment Scale (SDES). *Psychological assessment, 27*(4), 1182–1194. https://doi.org/10.1037/pas0000110

Martini, N. (2015). *Adaptación de las escalas de derrota y atrapamiento (defeat and entrapment) en población chilena: grupos clínicos y no clínicos en relación al estrés postraumático e intento suicida en accidentes laborales* (Tesis de Maestría). Universidad de Chile, Santiago de Chile.

Gross, J. J., & John, O. P. (2003). Individual differences in two emotion regulation processes: implications for affect, relationships, and well-being. *Journal of personality and social psychology, 85*(2), 348–362. https://doi.org/10.1037/0022-3514.85.2.348

Cabello, R., Salguero, M. J., Fernández-Berrocal, P., & Gross, J. J. (2013). A Spanish adaptation of the Emotion Regulation Questionnaire. *European Journal of Psychological Assessment, 29*(4), 234-240. doi: 10.1027/1015-5759/A000150

Nolen-Hoeksema, S., Larson, J., & Grayson, C. (1999). Explaining the gender difference in depressive symptoms. *Journal of personality and social psychology, 77*(5), 1061–1072. https://doi.org/10.1037//0022-3514.77.5.1061

Supplementary Table 2. Multiple comparisons between parameters (means and Odds ratios) according to profiles.

	Profile 1 (32%)	Profile 2 (34%)	Profile 3 (21%)	Profile 4 (11%)
		Symptoms of depression[a]		
Profile 1	-	< .001	< .001	< .001
Profile 2	-	-	< .001	< .001
Profile 3	-	-	-	< .001
		Symptoms of anxiety[a]		
Profile 1	-	< .001	< .001	< .001
Profile 2	-	-	< .001	< .001
Profile 3	-	-	-	< .001
		Symptoms of posttraumatic stress[a]		
Profile 1	-	< .001	< .001	< .001
Profile 2	-	-	< .001	< .001
Profile 3	-	-	-	< .001
		Suicide ideation (SI): Risk (≥ 3)[b]		
Profile 1	-	0.082 (0.016-0.428)	0.012 (0.003-0.055)	0.003 (0.001-0.016)
Profile 2	-	-	-	0.042 (0.024-0.072)
Profile 3	-	6.977 (4.116-11.824)	-	0.290 (0.186-0.450)
		Paranoid ideation: Sometimes (or more frequent)[c]		
Profile 1	-	0.052 (0.020-0.135)	.008 (0.003-0.020)	0.002 (0.001-0.006)
Profile 2	-	-	-	0.037 (0.020-0.068)
Profile 3	-	6.481 (4.311-9.745)	-	0.237 (0.125-0.450)
		Bizarre experiences (BE): Rarely[b]		
Profile 1	-	0.089 (0.056-0.141)	0.064 (0.038-0.109)	0.005 (0.000-0.074)
Profile 2	-	-	-	0.057 (0.004-0.876)
Profile 3	-	1.382 (0.703-2.718)	-	0.078 (0.005-1.292)
		Bizarre experiences (BE): Sometimes (or more frequent)[b]		
Profile 1	-	-	-	-
Profile 2	-	-	-	0.018 (0.010-0.034)
Profile 3	-	10.131 (5.691-18.034)	-	0.185 (0.115-0.298)

	Perceptual abnormalities (PA): Rarely[b]			
Profile 1	-	0.138 (0.087-0.217)	0.093 (0.059-0.145)	0.051 (0.030- 0.086)
Profile 2	-	-	-	0.372 (0.247-0.072)
Profile 3	-	1.484 (1.044-2.109)	-	0.552 (0.351-0.867)
	Perceptual abnormalities (PA): Sometimes (or more frequent)[b]			
Profile 1	-	0.128 (0.035-0.466)	0.038 (0.011-0.126)	0.013 (0.004-0.045)
Profile 2	-	-	-	0.103 (0.062-0.172)
Profile 3	-	3.386 (1.969-5.821)	-	0.349 (0.217-0.561)

[a]Profiles mean differences.

[b]ODDS Ratio and (95%CI); reference category: None.

[c]ODDS Ratio and (95%CI); reference category: None and rarely.

Supplementary Table 3. Multinomial Regression Parameters of Profile membership model

				95%CI	
	B	*SE*	OR	LL	UL
Low internalizing symptomatology (Profile 2)					
Cognitive reappraisal	0.14	0.09	1.14	0.95	1.37
Expressive suppression	0.33***	0.10	1.39	1.15	1.68
Rumination	1.93***	0.26	6.89	4.18	11.36
Entrapment	2.18***	0.50	8.86	3.03	23.76
Defeat	2.53***	0.48	12.55	4.86	32.43
Low internalizing & moderate post-traumatic stress symptomatology (Profile 3)					
Cognitive reappraisal	0.12	0.14	1.12	.85	149
Expressive suppression	0.14	0.15	1.15	.86	1.53
Rumination	3.75***	0.36	42.56	20.89	86.67
Entrapment	3.32***	0.58	27.52	8.91	85.06
Defeat	3.83***	0.52	45.94	16.63	126.90
At-risk (Profile 4)					
Cognitive reappraisal	0.32	0.22	1.37	0.89	2.13
Expressive suppression	0.29	0.21	1.35	0.90	2.03
Rumination	4.87***	0.42	130.15	56.22	301.28
Entrapment	4.57***	0.61	96.35	29.21	317.79
Defeat	5.06***	0.58	156.79	50.45	487.23

Note. N=1319. CI= confidence interval; LL= lower limit; UL= upper limit. OR=Odds ratio. Reference Profile= Without symptomatology (Profile 1).
*** $p < .001$; ** $p < .01$; * $p < .05$.
